# The cellular prion protein promotes neuronal regeneration after acute nasotoxic injury

**DOI:** 10.1080/19336896.2020.1714373

**Published:** 2020-01-17

**Authors:** Lindsay E. Parrie, Jenna A.E. Crowell, Julie A. Moreno, Stephanie S. Suinn, Glenn C. Telling, Richard A. Bessen

**Affiliations:** Prion Research Center, Department of Microbiology, Immunology and Pathology, Colorado State University, Fort Collins, CO, USA

**Keywords:** Cellular prion, neurotoxic injury, regeneration, differentiation, proliferation

## Abstract

Adult neurogenesis, analogous to early development, is comprised of several, often concomitant, processes including proliferation, differentiation, and formation of synaptic connections. However, due to continual, asynchronous turn-over, newly-born adult olfactory sensory neurons (OSNs) must integrate into existing circuitry. Additionally, OSNs express high levels of cellular prion protein (PrP^C^), particularly in the axon, which implies a role in this cell type. The cellular prion has been shown to be important for proper adult OSN neurogenesis primarily by stabilizing mature olfactory neurons within this circuitry. However, the role of PrP^C^ on each specific adult neurogenic processes remains to be investigated in detail. To tease out the subtle effects of prion protein expression level, a large population of regenerating neurons must be investigated. The thyroid drug methimazole (MTZ) causes nearly complete OSN loss in rodents and is used as a model of acute olfactory injury, providing a mechanism to induce synchronized OSN regeneration. This study investigated the effect of PrP^C^ on adult neurogenesis after acute nasotoxic injury. Altered PrP^C^ levels affected olfactory sensory epithelial (OSE) regeneration, cell proliferation, and differentiation. Attempts to investigate the role of PrP^C^ level on axon regeneration did not support previous studies, and glomerular targeting did not recover to vehicle-treated levels, even by 20 weeks. Together, these studies demonstrate that the cellular prion protein is critical for regeneration of neurons, whereby increased PrP^C^ levels promote early neurogenesis, and that lack of PrP^C^ delays the regeneration of this tissue after acute injury.

## Introduction

Adult neurogenesis occurs in a handful of brain regions, most notably the subgranular zone (SGZ) of the hippocampal dentate gyrus, the subventricular zone (SVZ) of the lateral ventricles, the olfactory epithelium of the nasal cavity, and most recently the amygdala [1–4]. Adult neurogenesis is comprised of several developmental processes, including progenitor cell proliferation, differentiation and maturation into a neuronal cell type, and formation of functional synaptic connections. Previous studies show a role for the cellular prion protein in these neurogenic processes [5]. Prions are perhaps best known for their role in transmissible spongiform encephalopathies including human Creutzfeldt-Jacob disease and cervid chronic wasting disease [6,7]. However, the role of the cellular form remains enigmatic, and is typically done in a mouse knockout background (see Wulf et al. [8], for review) and/or injury models such as oxidative stress or ischaemic damage [9–11]. From these studies, we know that PrP^C^ is important in pro-neurogenic processes, including the promotion of stem cell proliferation within the subventricular zone and differentiation of newly generated cells into a neuronal cell fate [5]. Others have reported that neurogenesis in the dentate gyrus can partially preserve hippocampal function during murine prion infection [12]. However, many studies report conflicting roles for PrP^C^ [8]. One confounding factor may be due to multiple adjacent cell types that may have differing functions of PrP^C^, thereby muting the effects [13]. Therefore, we use the olfactory system, which maintains its capacity to regenerate neurons via normal turnover well into adulthood [14]. This relatively isolated neuronal system, with just one neuron type in the olfactory sensory epithelium, consequently provides a unique and tractable model to study the role of PrP^C^ within an established neuronal network. Additionally, the olfactory mucosa has recently been identified as a safe, minimally invasive site for novel diagnostic analysis of human prion disease by real-time quaking-induced conversion (RT-QuIC) technology, underlying the importance of determining the function of PrP^C^ within this tissue [15,16].

Our previous *in vivo* study on the role of PrP^C^ in olfactory neurogenesis shows that the cellular prion protein promotes maintenance of mature neurons, thereby reducing OSN turnover [17]. However, under normal conditions, the effects of PrP^C^ on neurogenesis may be diluted out by the fact that developing neurons exist in a much larger population of mature neurons. To determine perhaps subtle PrP^C^ functions in neurogenesis, this study uses methimazole treatment. MTZ induces degeneration, and subsequent regeneration, of the entire olfactory epithelium while preserving the integrity of the lamina propria and cribriform plate, which are essential for OSN axon migration during re-innervation of the olfactory bulb (OB). Previous studies on the effect of MTZ-induced OSE injury demonstrate that the entire neuronal population is recovered, including axon re-innervation of the glomeruli in the OB, by 8 weeks post-injury (p.i.) [18].

This study examines the effect of PrP^C^ level on neurogenesis and survival after an acute injury in an *in vivo* system. By using methimazole to induce synchronized regeneration, we tease apart subtle differences of PrP^C^ level on neurodevelopment. Using congenic transgenic mouse models, we perform a temporal analysis on a large, discrete population of regenerating neurons. Using this model, we report the effects of PrP^C^ on the neurogenic processes of proliferation, differentiation and maturation, and axon targeting.

## Results

### PrP^C^ promotes rapid olfactory sensory epithelium regeneration

**Figure 1.**
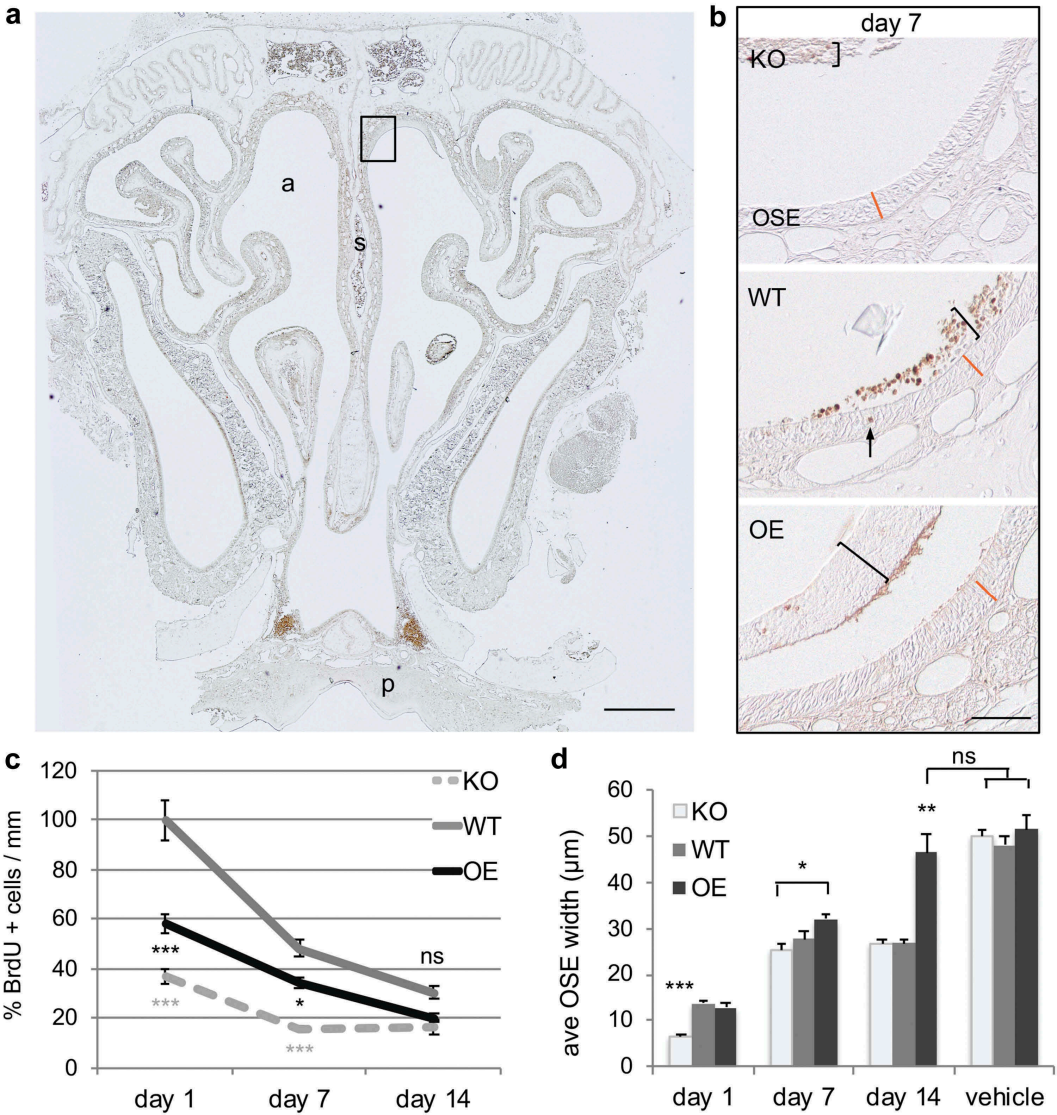
Cellular proliferation and survival of newly born cells in the regeneration of olfactory sensory epithelium are altered after acute nasotoxic injury. (a) Representative coronal section of nasal turbinate from animals that were collected at 1, 7, and 14 days after BrdU injection in both vehicle and MTZ treated animals. BrdU-labelling (brown stain) of mitotically active cells was performed to assess the effect of the prion level. a: nasal airway; s: septum; p: hard pallet. Scale bar is 500 μm. Dorsal septum region, boxed, is enlarged in panel (b): representative images of dorsal septum region quantified at day 7 in all three genotypes. Brackets highlight sloughed OSE retained within the nasal cavity, which is separate from the regenerating OSE. The arrow denotes a BrdU+ cell within the OSE. The orange line represents the WT OSE width at each time point, included in the OE and KO sections for visual comparison. Scale bar is 50 μm. (c) Proliferation was quantified at 1 day post-BrdU injection. BrdU-labelling at 7 and 14 days post-injection indicate survival. Quantifications are relative to WT day 1 values. (d) Regeneration of the OSE was quantified by measuring the OSE width at the dorsal septum in the posterior nasal cavity. Statistical analyses by ANOVA, where *P < 0.05, **P < 0.01, ***P < 0.001 (n = 3 each).

Transgenic FVB mice expressing altered levels of PrP^C^ were used to analyse synchronous neurogenesis after acute injury. Cellular proliferation, survival of newly generated cells, and OSE regeneration were analysed by immunohistochemistry (Figure 1). Animals were treated with MTZ to induce injury, and then injected the same day with the thymidine analogue BrdU to label mitotically active cells (brown stain Figure 1(a)). This protocol resulted in the loss of olfactory sensory epithelium throughout the entire nasal turbinate within 24 h, both in the anterior-posterior extent and medial-lateral extent (see Figure 1(b) for sloughed OSE). Quantification of BrdU-labelled cells along the nasal septum was performed in multiple tissue sections spanning the entire nasal turbinate for each animal. BrdU-labelling in PrP^C^ wildtype (WT) animals revealed a pattern comparable to normal adult proliferation, where a large population of proliferating cells is quickly lost between day 1 and 7 post-BrdU labelling (Figure 1(b)). The initial loss was 50% of cells, with an additional drop of 20% by day 14. In contrast to asynchronous adult regeneration, PrP^C^ knockout (KO) mice showed significantly less proliferation at day 1 (*P* < 0.001). Additionally, after the initial loss at day 7 (50%) the population stabilized, at lower levels than WT. Overexpression of PrP^C^ (OE) also displayed less BrdU labelling than WT, but also greater cell survival over time (66%). This was similar to what is seen under homoeostatic conditions, where a greater number of newly-born cells survived compared to WT.

Because the olfactory epithelium is sloughed off after MTZ treatment, and begins to regenerate within 24 h, it is possible to look at the amount of regeneration over time. Quantification of maximal tissue regeneration at day 1 showed that there is an average thickness of 14.3 μm in PrP WT animals (Figure 1(a,c)). This was also the same thickness in the OE animals (14.26 μm). However, OSE regeneration was significantly reduced in KO, with an OSE thickness of 6.73 μm (approximately one cell layer). This data shows a dramatic delay in regeneration in the absence of PrP^C^. At 7 days after injury, the average OSE thickness was 3.01 μm in WT, 28.65 μm in KO, and 34.53 μm in OE animals. Interestingly, by day 14, WT and KO OSE regeneration had both plateaued, however not to vehicle-treated levels, with equivalent thicknesses of 28.29 μm and 28.76 μm, respectively. OSE width in OE animals had recovered to vehicle-treated levels by day 14 post-injury, with no statistical difference between 49.95 μm and 54.5 μm. Together, this data shows that PrP^C^ promotes regeneration of epithelial tissue after acute injury, with overexpression resulting in even greater recovery than wild type levels.

### Neuronal differentiation is sensitive to PrP^C^ dose

**Figure 2.**
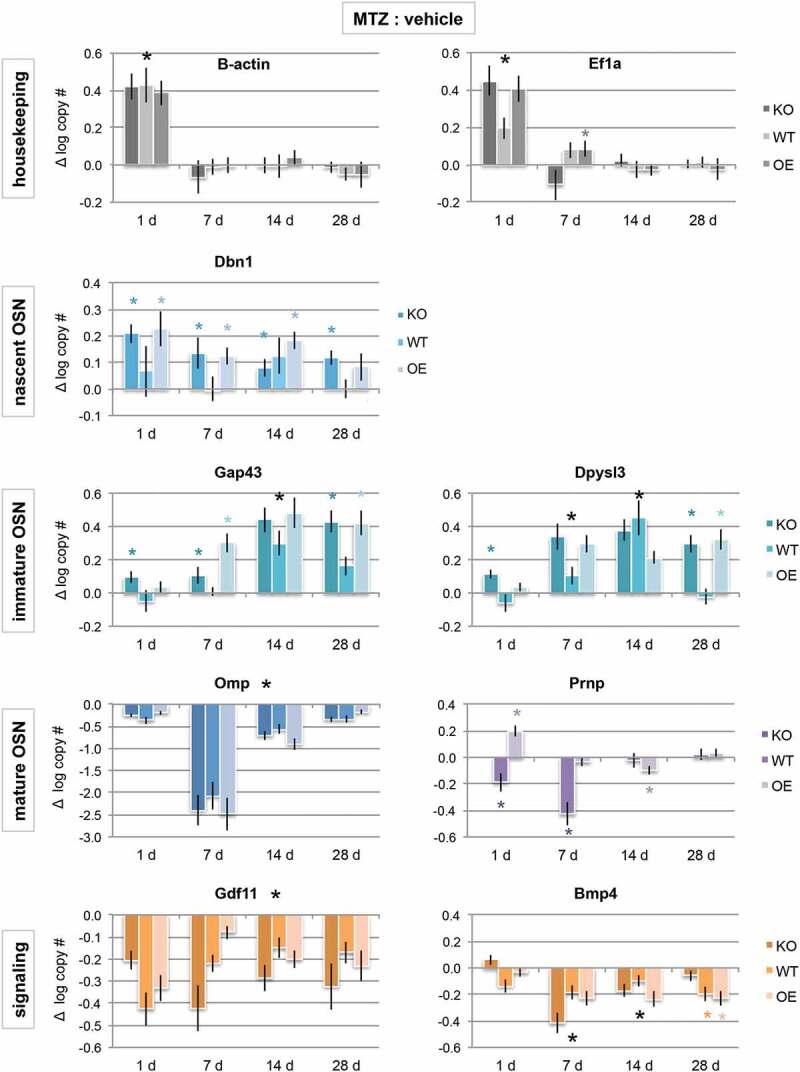
Cellular prion promotes early neuronal differentiation. mRNA expression levels of house-keeping genes (grey bars), developmental stage-specific markers (blue bars), and signalling genes that promote or inhibit neuronal maturation (orange bars) by quantitative PCR. Purple bars are prion (Prnp) gene expression, which span immature to mature OSN timepoints. Data are presented as a difference in total log gene copy number of MTZ to vehicle expression ± S.E. of the difference. Statistical comparison was done by ANOVA, where * *P* < 0.05; large black * = significant difference observed in all genotypes, while small coloured * = observed in only one or two genotypes (KO n = 8 vehicle, MTZ n = 8; WT n = 8 vehicle, n = 6 MTZ; OE n = 8 vehicle, n = 10 MTZ).

To determine whether differentiation of newly regenerated olfactory epithelial cells was altered between the PrP genotypes, expression of developmental stage-specific markers was analysed by quantitative PCR (qPCR, Figure 2). Under normal, asynchronous regeneration, newly-born cells express drebrin1 (Dbn1) and will begin differentiating into immature neurons between 2–7 days and into mature OSNs by 7–14 days [19–21]. In the olfactory epithelium, the progression from the immature OSN (characterized by Gap43 and Dpysl3 expression) to mature OSN phenotype expressing olfactory marker protein (OMP) is inhibited by the morphogen BMP4 [22]. Once the mature neuron dies, this stimulates proliferation through dis-inhibition of GDF11 [23].

Quantification of these developmental stage-specific genes after injury showed some surprising responses. First, the expression of typical house-keeping genes β-actin (or Actb) and EiF1a were both greatly increased as compared to the vehicle just 1 day after injury (*P*< 0.05). However, house-keeping gene expression levels returned to normal levels by day 7, indicating a large increase in rebuilding and transcription immediately after the injury that became more measured within 1 week. These data also highlight the importance of analysing total mRNA copy number, as opposed to normalizing to housekeeping genes, as expression level changes can vary gene to gene.

Early neuronal gene expression in animals with wild type PrP^C^ levels showed no significant increases as compared to the vehicle until days 7 to 14 post-injury (Dbn1, Gap43). Together with the proliferation/survival data (Figure 1), this indicates that proliferation did not lead to differentiation until days 7–14 in WT animals. As opposed to WT, the absence of PrP^C^ results in an immature phenotype for longer (Dbn1, Gap43, Dpysl3), indicating a delay in recovery after injury. Overexpression of PrP^C^ also resulted in increased nascent and immature gene expression (Dbn1, Gap43, Dpysl3). When combined with the regeneration data (Figure 1), in which there was a greater amount of tissue at these time points, this indicates that the population is regenerating more quickly and nearly recovered to vehicle-treated levels (which always have greater immature levels than WT or KO, likely due to inhibition of maturation by BMP4 [17]). Although mature OSN expression (OMP) is significantly reduced in all genotypes, if gene expression were examined at 42 days post-injury, it would be possible that expression levels in PrP OE animals would be stabilized, while WT would require additional time to recover to vehicle-treated levels (which would coincide with 8 weeks others have previously reported [18]), and KO may take even longer, or never stabilize. Taken together, these data indicate that PrP^C^ is important for differentiation into mature OSNs, and that lack of PrP^C^ creates deficiencies in the last maturation steps.

**Figure 3.**
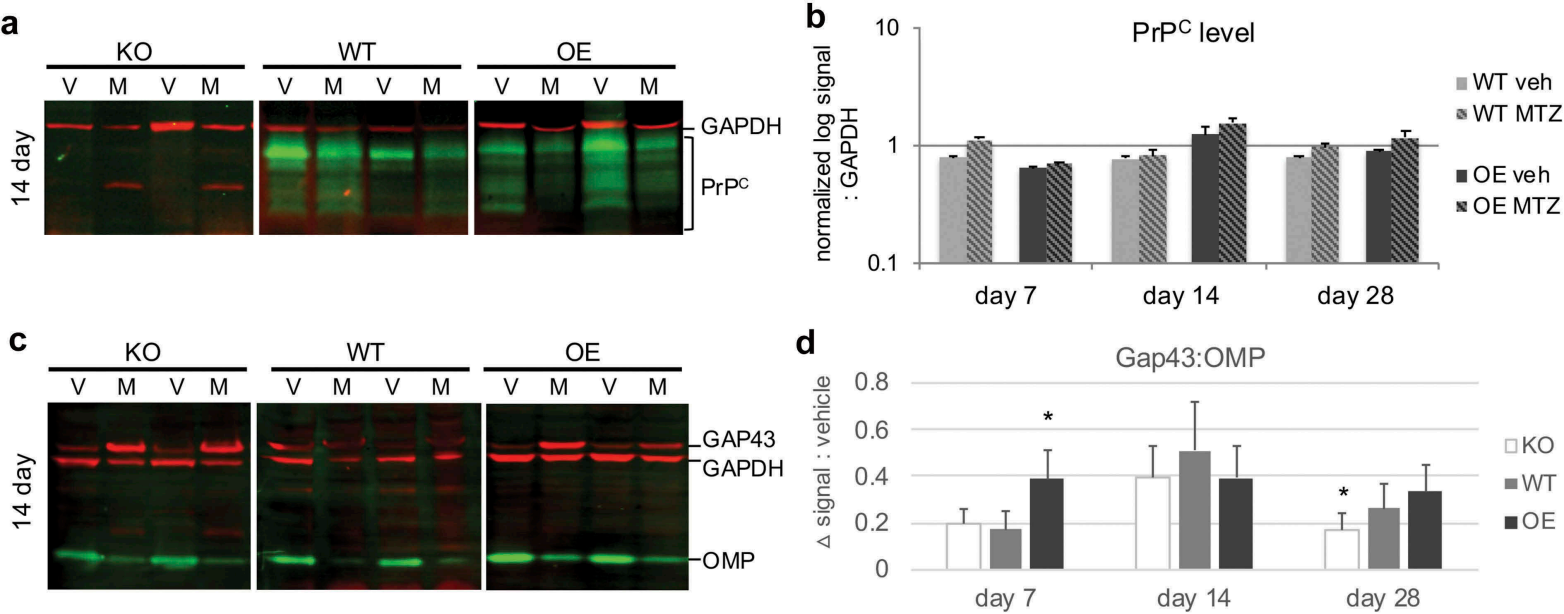
Influence of prion dose on protein levels after injury. Olfactory tissues were analysed by multiplex western blot. (a) Representative immunoblot at day 14 of PrP^C^ protein levels (green) after vehicle (V) or methimazole (M) treatment. GAPDH loading control was visualized in red. (b) Quantification of PrP^C^ at 7, 14, and 28 days after drug treatment, where log-transformed PrP^C^ signal was normalized to GAPDH. Data were analysed by Student’s *t*-test (MTZ:vehicle, *P < 0.05, n = 5 each treatment). (c) Representative day 14 multiplex immunoblot of canonical olfactory neuron maturation markers: immature olfactory sensory neuron marker GAP43 (red, 43 kDa), mature OSN maker OMP (green, 20 kDa) and loading control GAPDH (red, 37 kDa). (d) Quantification of the difference in average relative maturation signal (GAP43:OMP) after injury as compared to vehicle levels ± S.E of the difference between the means. Data were analysed by two-way ANOVA (*P < 0.05, n = 5 each treatment).

One final expression trend of note was the difference in prion mRNA (Prnp) expression between WT and OE (there was no detectable Prnp expression in KO animals, Figure 2). Prnp is expressed in GAP43 and OMP-expressing OSE cells, but not nascent OSNs [24], indicating that expression begins in the immature neuron and is maintained in mature neurons. After MTZ-induced injury, WT animals showed a significant decrease in Prnp mRNA levels as compared to vehicle at both 1 and 7 days, most likely due to loss of the immature and mature neurons. Prnp expression returned to vehicle levels by 14 days. Interestingly, there was a significant increase in Prnp expression in PrP OE animals at 1-day post-injury, which then appeared to self-correct by day 14 and return to vehicle levels at day 28. To further examine these expression trends, we quantified protein levels by fluorescent multiplex immunoblot at days 7, 14, and 28 post-injury (Figure 3).

PrP^C^ protein levels after injury were compared to vehicle levels within each blot (Figure 3(a,b)). There were no statistical differences between MTZ and vehicle for either PrP-expressing genotype, although day 7 and day 28 in WT were trending towards higher PrP^C^ levels after injury (*P* = 0.07 and 0.06, respectively). The observed differences may be due to the relative sensitives of each assay, the time point chosen as opposed to the relative timing of transcription/translation, and/or different stabilities of the mRNA versus protein.

The relative OSN differentiation status was also explored by immunoblot (Figure 3(c,d)), where the difference in immature:mature neuron levels between MTZ and vehicle were compared across all three PrP genotypes. For all three PrP genotypes, there was a significant shift in the OSN population to a more immature phenotype at all time points, consistent with a tissue undergoing regeneration. As opposed to normal adult neurogenesis, which sees neurons differentiating into immature neurons between 4 and 8 days, peak immature status appeared to be reached at day 14 in WT and KO. Interestingly, lack of PrP^C^ showed a quicker reversion to vehicle maturation levels, despite a smaller OSE width, indicating a less robust recovery. However, consistent with our regeneration and proliferation data, PrP OE cells recovered more quickly (peak immature status at day 7), but also maintained a regenerating phenotype by day 28. Taken together, these data reveal subtle effects of PrP^C^ dose, and its importance in regulating the timing of recovery.

### Axon targeting in methimazole treated animals does not recover

**Figure 4.**
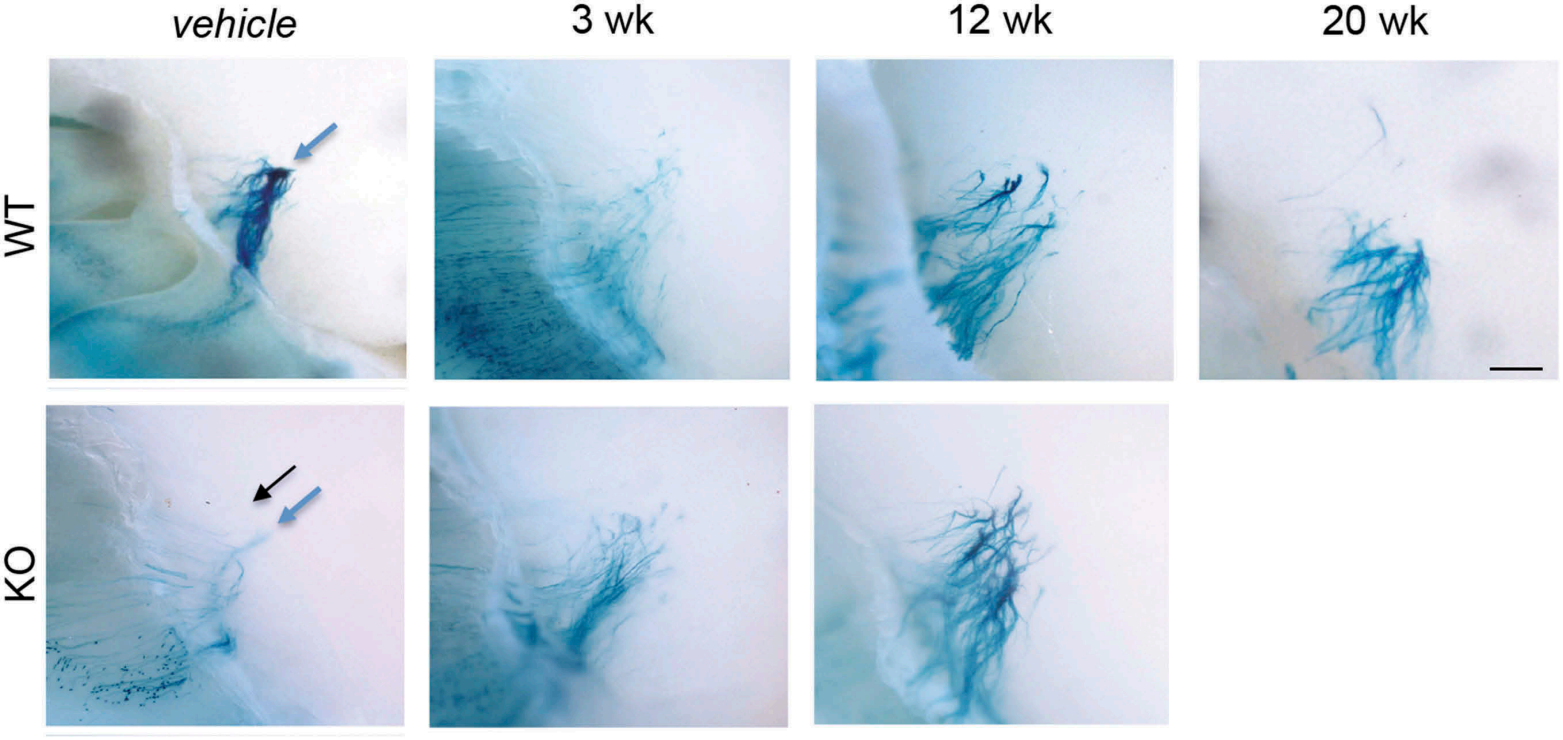
OSN axon targeting does not recover by 20 weeks post-MTZ treatment. Visualization of β-lactamase expressing transgenic olfactory neuron axons in PrP WT and KO olfactory bulb glomerular layers. After vehicle or methimazole treatment at 10 weeks of age, animals recovered from 3 to 20 weeks, at which time tissues were collected and stained with X-gal to visualize the entire P2 OSN cell, from soma to axon terminal. Normal axon targeting to glomeruli for each PrP^C^ genotype shown in vehicle panels (blue arrows: full glomeruli, black arrows: ectopic). Axon mistargeting persisted after MTZ treatment in both genotypes. Scale bar represents 500 μm.

One proposed role for PrP^C^ is in neurite outgrowth [25–27], and our previous studies showed PrP^C^ is important in glomerular targeting of OSN axons [17]. Transgenic mice expressing altered levels of PrP^C^ were used to analyse axon targeting of a particular OSN expressing the P2 olfactory receptor. X-gal staining of these P2 neurons highlights the entire neuron from cell bodies in the olfactory epithelium to axon termini in the glomerular layer of the olfactory bulb (Figure 4). Under normal conditions, ~2.5 glomeruli are targeted in WT animals, while there are increased glomerular offshoots in PrP KO (Figure 4, and Parrie et al. [17]). The current data do not support previous studies, which show that glomerular targeting by OSN axons is abolished after MTZ or other injury methods, but critically, retargeting is completely refined to vehicle-treated levels by 8 weeks post-MTZ treatment [18,20].

## Discussion

The biological function of the cellular prion protein has been extensively investigated, but the various, potentially conflicting, roles for PrP^C^ in neurogenesis remain to be clarified. In adult neurogenesis of both the subventricular zone and dentate gyrus, PrP^C^ expression increases cellular proliferation as compared to PrP knockout mice [5]. However, the final number of neurons in the dentate gyrus is unchanged between PrP genotypes, indicating that while PrP^C^ is important in proliferation, it is not essential for neuronal differentiation in the hippocampus. Alternatively, other studies demonstrate a pro-glial role for PrP^C^, at the expense of cell proliferation [28]. Contradictory studies also demonstrate that PrP^C^ either inhibits [29] or promotes neurite outgrowth [30]. These differing effects may be explained in part by differences in experimental conditions, such as line of knock-out animal used or *in vitro* vs. *in vivo* analysis.

Here we compared neurogenesis *in vivo*, using congenic wild type, null, and over-expressing PrP mice to discover that PrP^C^ influences neuronal epithelium regeneration. We present evidence that the cellular prion protein promotes adult neurogenesis during synchronous regeneration. Using temporal analysis of neurogenic processes, we also illustrate the requirement of PrP^C^ for normal precursor proliferation and survival. Moreover, precise PrP^C^ dose is critical for the correct timing of OSN differentiation. Taken together, our results highlight the proneuronal functions of PrP^C^ after acute injury.

Comparable to our previous studies, PrP^C^ promotes survival of newly born olfactory neurons [17]. In-depth mRNA expression analysis of the newly regenerated cells reveals that PrP^C^ promotes precursor differentiation into a neuronal cell fate quickly, but that a homoeostatic state has not yet been reached by 28 days post-injury, regardless of PrP genotype or levels. Because there are still sloughed cells within the nasal cavity at 1 day after injury, it may be possible that some expression changes are obscured, which may account for the observed increase in Prnp levels of the over-expressor (OE) group as well as the delayed decrease in levels of the mature neuron marker OMP until day 7. However, because we observe significant changes in nascent gene expression and tissue regeneration by day 1, it is unlikely that observed levels are due solely to remnant tissue.

Interestingly, the trend of less significant changes in wild type differentiation-specific genes as compared to KO and OE signifies that differentiation is exquisitely sensitive to Prnp dose. Overexpression appears to increase expression of nascent and immature markers and nearly recovers a normal mature status by the 28-day timepoint. Further investigation at 32 days post-injury could definitively determine whether PrP overexpression results in faster recovery to a normally differentiated OSN population.

Lack of PrP^C^ expression results in prolonged proliferation and differentiation expression, but also deficiencies in the final maturation steps. For example, decreased levels of the inhibitory genes Gdf11 and Bmp4 should facilitate the division of neural precursor cells [31] and mature OSN differentiation [32] combined with increase nascent OSN gene expression. However, the continued elevated expression of early markers, together with the lower BrdU proliferation rate and GAP43:OMP ratio by immunoblot at later timepoints, indicate an inability to generate and properly maintain new OSNs in cells lacking PrP^C^. This phenomenon has been observed in previous studies. Regeneration after a mechanical injury is attenuated in PrP KO cells, and there is a delay in myogenic precursor cells exiting the cell cycle [33]. Oligodendrocyte precursor cells of PrP KO pups also demonstrate altered proliferation over time and delays in differentiation [28]. In neurons, lack of PrP^C^ results in prolonged cellular proliferation and maturation which ultimately does not change adult cell numbers in either the cerebellar granular layer or the dentate gyrus [5,26,28]. For the present study, a second round of BrdU treatment at one or more of the later time points would be able to determine if there is a sustained increased proliferation in KO as compared to WT, and whether this leads to altered numbers of mature neurons over time.

The final stage of adult neurogenesis is the integration into existing neuronal circuitry, in this case in the olfactory bulb. The projection of axons and formation of functional synaptic connections are essential for stabilizing OSN survival [20] and glomeruli are fully mature by the end of the second postnatal month [34]. The prion protein has been shown to regulate mechanisms of axon formation, including axon initiation, growth cone development, and neurite outgrowth [25–27,35,36]. After MTZ-induced injury, previous work [18] demonstrates complete axon regeneration and glomerular refinement by 8 weeks p.i. under identical experimental conditions as described here. However, we were unable to replicate these findings, and because mistargeted OSN axons did not refine even in PrP WT olfactory bulbs, it is impossible to ascertain the effect of PrP^C^ on targeting after acute injury. Although axon targeting does not recover to wild type levels, the axon paths do become stronger, the olfactory epithelium does regenerate, and gene expression shows OSN maturation in all genotypes, together indicating a type of functional recovery that has stabilized. Previous evidence supports these findings. PrP null mice are capable of differentiating scents despite deficiencies in signalling [37] and axon targeting [17].

Further work would be necessary to optimize MTZ treatment to carefully control for the effect of PrP^C^ dose on axon targeting after injury. Alternatively, dexamethasone (DXM) treatment after MTZ-induced injury may enhance recovery [38], and provide a better system to investigate axon targeting. Additionally, other factors may influence axon targeting at the synaptic side. Therefore, future investigation into the status of the OB mitral/tufted cell dendrites after injury would be interesting [39], although homotypic OSN axon convergence is not dependent on the presence of the olfactory bulb [40]. Investigation into the role of PrP^C^ in the specific mechanisms of *in vivo* axon targeting, whether in neurite outgrowth or local glomerular specification, is needed.

## Materials and methods

### Animals and methimazole treatment

Male and female congenic mice on an FVBCr/N background, including wildtype, PrP-KO (Zurich I line), and PrP-OE (Tg4112^+/+^) mice were used for proliferation and differentiation studies (from the Prion Research Center, Colorado State University). For axon tracing, B6;129P2-Olfr17^tm1Mom^/MomJ mice (Jackson Laboratories, Bar Harbor, ME) were used. To induce synchronized regeneration, 10-week-old animals were treated by intra-peritoneal (i.p.) methimazole injection, at 100 mg/kg body weight as described previously [41]. All animals were bred and maintained by the laboratory animals in research (LAR) facility and all protocols were approved in accordance with the Colorado State University IACUC committee guidelines.

### BrdU treatment and tissue collection

Quantification of cell proliferation and survival in the OSE was done as previously described [17]. Briefly, the thymidine analogue 5ʹ-bromodeoxyuridine (BrdU) (Sigma-Millipore, St. Louis, MO) was injected 2 h after MTZ lesioning. BrdU was administered as three pulse labels (three i.p. injections, 2 h apart; 50 mg/kg of body weight, in sterile saline). Animals were perfused 1, 7, or 14 days after the last BrdU administration. These collections correspond with developmentally important time points and were used to determine the proliferation and survival of newly born OSNs. Animals were deeply anaesthetized with Isoflurane (MWI Veterinary Supply, Boise, ID) and were transcardially perfused with 0.1M PBS followed by periodate-lysine-paraformaldehyde (PLP) fixation [42]. Nasal turbinates were collected whole, postfixed in PLP 24 h before decalcification in 10% formic acid and paraffin embedding. Coronal sections were cut at 4 μm thick using standard methods.

### Histology

Histological procedures were done as described in Parrie et al. [17]. Briefly, all immunohistochemical procedures were performed on a minimum of every sixth tissue section to prevent quantification of the same region across adjacent sections. Six slides spanning the entire nasal turbinate were quantified for each animal to allow for complete analysis. For BrdU staining, antigen retrieval was completed in 1M HCL for 15 min at 37°C. Blocking was completed in a series of 20% Streptavidin/10% normal donkey serum (NDS), 20%Biotin/10%NDS, and 20 μg/mL Donkey anti-Mouse (Fab) Ig fragments (Jackson Immuno-Research Laboratories, West Grove, PA, AntibodyRegistry: AB_2307338) in TBST. The primary antibody (Bu20a mouse anti-BrdU monoclonal, 1:400; Cell Signalling Danvers, MA, AntibodyRegistry: AB_2307338) was applied overnight at 4°C and incubation in the secondary antibody (donkey-anti-mouse-biotinylated, 3 μg/ml; Jackson ImmunoResearch, AntibodyRegistry: AB_2307438) at room temperature for 1 h. Streptavidin-horseradish peroxidase (HRP) (Biosource International, Camarillo, CA) was used at 1:800 and incubated at room temperature for 20 min. No counterstain was performed in order to visualize nuclear BrdU staining in the densely packed OSE. BrdU was then visualized with localization of HRP activity with DAB+ chromogen (Dako, Carpinteria, CA), quantified on a Nikon Eclipse E600 microscope using DIC and tissue morphology for structural localization. OSE length measurements were obtained using an Olympus camera/CellSens standard software and analysed as described in Image Analysis below.

### Quantitative real-time PCR

All procedures were done as described in Parrie et al. [17]. Total RNA was isolated from nasal turbinates using TRIzol reagent (Invitrogen) according to the manufacturer’s protocol. For PrP WT and KO genotypes, RNA was isolated from 6/8 vehicle and 8 MTZ treated animals and for PrP OE, n = 8 vehicle and 10 MTZ. Total RNA concentration was determined by absorbance at 260 nm and 3.5 μg was used for 20 μl cDNA synthesis reactions using AMV reverse transcriptase (Fisher Scientiﬁc) and Oligo(dT)12–18 primer (Life Technologies) as described previously [17,43]. Resulting cDNA was treated with RNase H (New England Biolabs, Ipswich, MA) to remove complementary RNA. Quantitative PCR analysis was conducted using BioRad SsoFast™ EvaGreen® Supermix and CFX96 thermal cycler. Triplicate 20 μl reactions each consisted of 1 μl ﬁrst-strand cDNA, 10 μl 2x EvaGreen® Master Mix, 0.25–1 μl each of forward primer and reverse primer (at 10 mM starting concentration), and 7 μl PCR grade water. PCR conditions included an initial denaturation at 95°C for 30 s, followed by 35–40 cycles of denaturation at 95°C for 7 s and annealing/extension at 59–62.5°C for 20 s. At the end of each run, an analysis of the PCR product melting temperature was conducted. Amplicon speciﬁcity was also veriﬁed by sequencing. Transcript concentrations were calculated with BioRad software using standard curves produced by serial dilution of puriﬁed PCR product (1 ag/ml to 10 ng/ml) which were conﬁrmed by sequencing. Statistical analysis was performed on log-transformed means for transcript abundance that compared MTZ to vehicle animals by Student’s *t*-test. All data are presented as the difference between means ± S.E. of the difference between means, with the level of signiﬁcance set at *P*< 0.05.

Primers were designed for the following genes:

*Actin, beta* (GenBank Accession NM_007393, F: 5ʹ-TGT GAT GGT GGG AAT GGG TCA GAA-3ʹ, R: 5ʹ-TGT GGT GCC AGA TCT TCT CCA TGT-3ʹ); *Bmp4* (GenBank Accession NM_007554, 48F: 5ʹ-ATC AGG AGA TGG TGG TAG AG-3ʹ, 49R: 5ʹ-AGT TTG TGT GGT ATG TGT AGG-3ʹ); *Dbn1* (Genbank Accession NM_001177371, F: 5ʹ-TTT GAA CAG GAA CGG ATG G-3ʹ, R: 5ʹ-ACC TCC TCT TGG CTT CTT-3ʹ); *Dpysl3* (GenBank Accession NM_009468, F: 5ʹ-AAG GAA ATG TGG TCT TTG GCG AGC-3ʹ, R: 5ʹ-TGG CCA GCA AGG AGT TGA TGT AGT-3ʹ); *Eif1a* (GenBank Accession NM_010120, F: 5ʹ-CAA CAC TGT TTG CTG CCT GTG GAT-3ʹ, R: 5ʹ-ACA GCA GCT GAG ACT CCT TTC CAA-3ʹ); *Gap43* (GenBank Accession NM_008083, F: 5ʹ-ATA AGG CTC ATA AGG CTG CGA CCA-3ʹ, R: 5ʹ-TCC TTC TTC TCC ACA CCA TCA GCA-3ʹ); *Gdf11* (GenBank Accession NM_010272.1, F: 5ʹ-TCA GCC GGG AGG TAG TGA AG-3ʹ, R: 5ʹ-GGT AGC GTG GTA CTC GTC CT-3ʹ); *Omp* (GenBank Accession NM_011010, F: 5ʹ-ATT GAG CTG GTA CTG GCT TGT GGA-3ʹ, R: 5ʹ-AAA TGC CAG CCT TCC TAA CAG GGA-3ʹ); *Prnp* (GenBank Accession NM_011170, F: 5ʹ-CAT GAG CAG GCC CAT GAT CCA TTT-3ʹ, R: 5ʹ-TGC ACG AAG TTG TTC TGG TTG CTG-3ʹ).

### Western blot analysis

Tissue collection and western blot were performed as described previously [17,44,45]. Briefly, 40 μg of 10% nasal turbinate homogenates was used for the analysis of PrP^C^ (D18 at 1:5000, produced in house), GAP43 (rabbit-anti-Gap43 antibody at 0.4 μg/μl) and OMP (goat-anti-OMP polyclonal antibody, Wako Chemicals, Richmond, VA at a 1:5000 dilution). For detection of GAPDH loading control, chicken-anti-GAPDH antibody (EMD Millipore) was used at 0.33 μg/μl. The secondary antibodies were IRDye conjugates available from LI-COR (Lincoln, NE): 800CW Gt Anti-Human IgG for detection of D18, 800CW Dky Anti-Goat IgG, 680RD Dky Anti-Rabbit IgG, and 680RD Dky Anti-Chicken IgG, all at concentrations of 0.02 μg/ml. Blots were visualized on the Odyssey CLx Imager system (LI-COR) and associated Image Studio 4.0 software was used to measure the total signals of each protein. For PrP^C^ analysis, signal levels were log-transformed for comparison purposes and normalized to the GAPDH signal within each lane. Quantification of the relative differentiation status was performed by comparing the GAP43:OMP signal ratio of methimazole treated samples to the vehicle for each time point. Statistical analysis was performed using Student’s *t*-test (vehicle:MTZ), with *P* values of <0.05 considered significant.

### Visualization of P2 OSN axons

Male and female B6;129P2-Olfr17^tm1Mom^/MomJ (also known as P2-IRES-tau-lacZ mice, available from the Jackson Laboratory) on a mixed C57BL/6 129 background were collected after drug treatment (WT n = 2 MTZ and 3 vehicle at 3 wks; 7 each MTZ and vehicle at 12 wks; 7 each at 20 wks. KO n = 2 each MTZ and vehicle at 3 wks; 8 MTZ and 7 vehicle at 12 wks). Wholemount processing was carried out as described previously [17,46]. The skull, including nasal tissue and anterior half of the brain, was immersion fixed in ice-cold 4% paraformaldehyde for 2 h at 4°C. The tissue was then decalcified for 24–48 h in 14% ethylenediaminetetraacetic acid (EDTA) tetrasodium solution at 4°C before wholemount X-gal staining was performed as described in [17]. Whole mounts were examined using a Nikon SMZ800 dissecting microscope and multidimensional images were captured using the Instant EPI module in cellSens Standard software on a BX51 Microscope with DP70 CCD camera (Olympus).

### Image analysis

Images were analysed for OSE length or width using ImageJ (Rasband, W.S., Image J, National Institutes of Health, Bethesda, Maryland, USA http://rsb.info.nih.gov/ij/, 2015). For BrdU analysis, the lengths of the septal OSE were measured using the free line drawing tool in ImageJ to determine the number of mitotic cells per mm of OSE. To identify a consistent region for width analysis across animals, nasal turbinates in tissue cross sections 21 and/or 22 (as described in Mery et al. [47]) were imaged with 20x magnification on the Olympus BX51 Microscope with DP70 CCD camera. To quantify the maximal regeneration in each genotype, the widest portion of the OSE within the most dorsal 2 mm of the septum was measured using the line drawing tool in Image J. The average widths from each genotype (KO = 16 veh, 28 MTZ; WT = 23 veh, 26 MTZ; OE 18 veh, 28 MTZ sections per time point) were statistically compared by ANOVA with Tukey post-hoc comparison and significance at *P*< 0.05.
